# Colorectal infantile myofibromatosis: an unusual cause of rectal prolapse and sigmoid colo-colonic intussusception: a case report

**DOI:** 10.1186/1757-1626-1-397

**Published:** 2008-12-15

**Authors:** Deepti Dhall, Philip K Frykman, Hanlin L Wang

**Affiliations:** 1 Department of Pathology and Laboratory Medicine, Cedars-Sinai Medical Center, Los Angeles, California, USA; 2 Division of Pediatric Surgery, Department of Surgery, Cedars-Sinai Medical Center, Los Angeles, California, USA

## Abstract

**Background:**

Infantile myofibromatosis is a rare mesenchymal disorder of infancy that can extensively involve the viscera including the gastrointestinal tract.

**Case presentation:**

In this report, an exceptional case of infantile myofibromatosis is described in which rectal prolapse and sigmoid colo-colonic intussusception were the initial presentations of colorectal involvement in a 2-month-old premature female infant.

**Conclusion:**

To the best of our knowledge, this is the first case reporting rectal prolapse and the second case documenting intussusception secondary to gastrointestinal involvement by infantile myofibromatosis.

## Background

Infantile myofibromatosis (IM) is a rare mesenchymal disorder that can be solitary, multicentric or generalized [1]. Visceral involvement occurs in 40% of generalized cases [2]. The gastrointestinal tract is a common site of visceral involvement and the most common clinical presentation is diarrhea. Rare cases with intestinal obstruction or perforation have also been described [3-6].

In this report, we describe an exceptional case of IM that occurred in a premature infant with multiple skin and soft tissue nodular lesions. Her colorectal involvement was discovered due to the occurrence of rectal prolapse and sigmoid colo-colonic intussusception.

## Case presentation

**Figure 1:**
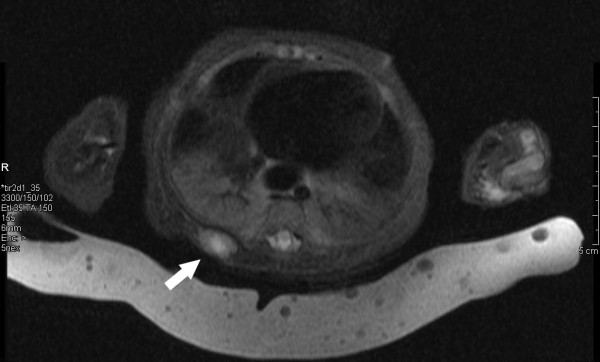
Magnetic resonance imaging of the right posterior hemithorax showing a soft tissue lesion. The nodule was inferior to the level of the scapula and was within the paraspinal muscles measuring 1.5 × 0.9 × 1.8 cm.

The patient was a 2-month-old premature infant who was twin A of a set from fraternal twin girls, and was born at 32 weeks of gestation age via Cesarean section to a 33-year-old gravida 2, para 0 woman who underwent an *in vitro *fertilization pregnancy for a bicornuate uterus. While in the neonatal intensive care unit (NICU), the baby was found to have multiple nodules on her abdomen, chest and extremities, the largest measuring 1.8 cm on greatest dimension. Head, thoracic, abdominal and pelvic magnetic resonance imaging revealed the nodules to be primarily in the skeletal muscle and subcutaneous tissue. In addition, a 5 mm metaphyseal enhancing lesion was noted in the right proximal femur. Incisional biopsy was performed on the large nodule shown in Figure 1 to obtain a tissue diagnosis.

During her NICU course, she also had frequent heme-positive stools but no feeding intolerance or clinical signs of bowel obstruction. She presented with rectal mucosal prolapse. A 5 mm polypoid lesion was noted in the rectum while the prolapse was treated by submucosal injection of 50% dextrose solution as a sclerosing agent. The patient presented one day later with what appeared to be recurrent rectal prolapse that was manually reduced. To further evaluate her gastrointestinal tract, a barium enema was performed, which revealed a colo-colonic intussusception localized to the sigmoid colon that was not reducible by hydrostatic technique, laparoscopic maneuver, or manual reduction. The patient underwent a sigmoid colectomy with primary anastomosis. A barium enema of the entire colon 3 months after resection did not identify any additional lesions. Her postoperative course was uneventful and she has been doing well for the past 6 months after the surgery.

## Pathologic findings

**Figure 2:**
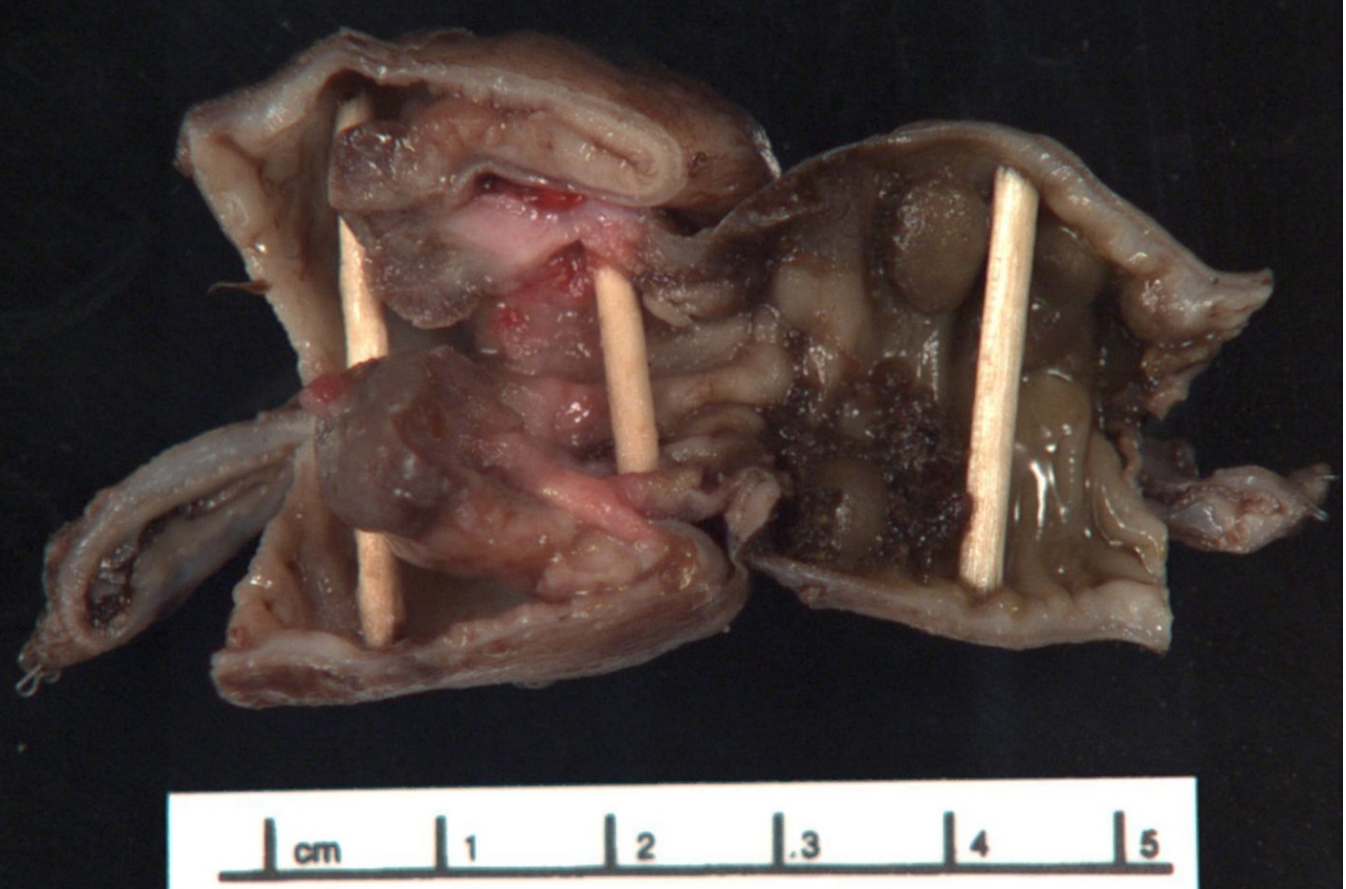
Sigmoid colon resection specimen showing polypoid lesions, one of which led the intussusception.

**Figure 3:**
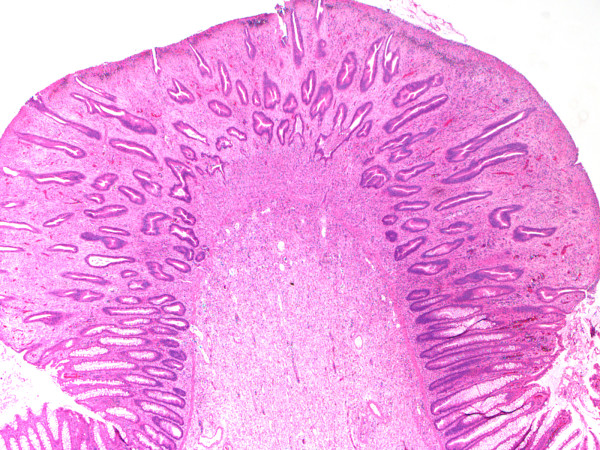
Low-power view of a polypoid lesion identified in sigmoid colon resection specimen showing irregular glands, expansion of lamina propria and surface erosion (hematoxylin-eosin stain, original magnification ×20).

**Figure 4:**
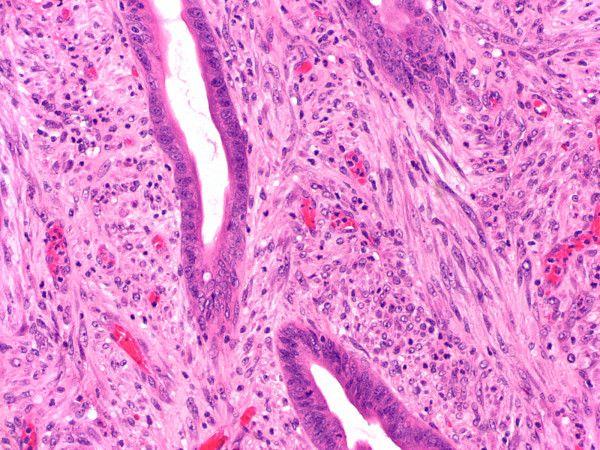
Higher power view of the lesion showing irregularly arranged bland spindle cells in the lamina propria (hematoxylin-eosin stain, original magnification ×200).

**Figure 5:**
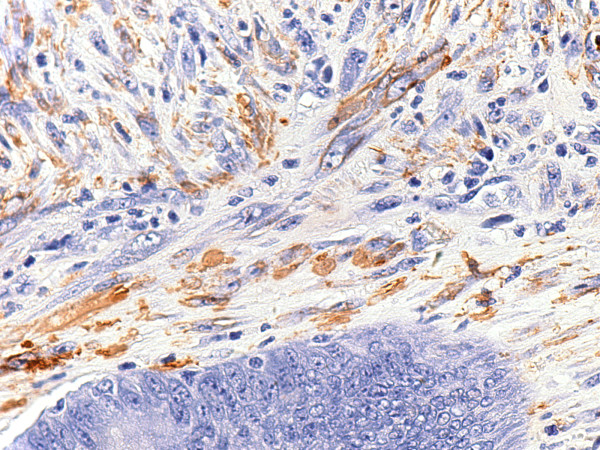
Lamina propria spindle cells expressing smooth muscle actin (immunohistochemistry, original magnification ×400).

The sigmoid resection specimen consisted of a 6 cm segment of large intestine with a 2 cm intussusception (Figure 2). Six polypoid nodules were identified on the mucosal surface, ranging in size from 0.3 to 1.7 cm in greatest dimension. Microscopically, these nodules displayed a bland spindle cell proliferation of variable cellularity, predominantly seen in the mucosa with only focal disruption of the muscularis mucosae and focal extension into the superficial submucosa (Figure 3). Superficial surface erosions were also evident. The spindle cells expanded the intercrypt spaces to replace the lamina propria with only scattered admixed inflammatory cells. No mitotic figures were identified in spindle cells. The crypts appeared to maintain their orderly arrangement, but were distorted, irregularly shaped and somewhat compressed (Figure 4). The epithelial cells exhibited reactive and regenerative changes with frequent mitotic figures. Immunohistochemically, the spindle cells stained positive for vimentin and smooth muscle actin (Figure 5), and negative for desmin, S100, CD34 and ALK-1, consistent with a myofibroblastic origin.

The biopsy of the subcutaneous nodule consisted of a 1.0 × 0.8 × 0.7 cm and a 1.4 × 0.6 × 0.2 cm white-tan soft tissue fragments that also showed myofibroblastic proliferation histologically. The myofibroblasts were plump with modest eosinophilic cytoplasm and arranged in irregular fascicles, reminiscent of nodular fascitis. There was no significant nuclear atypia or mitotic activity. Occasional inflammatory cells and multinucleated giant cells were present.

Given the clinical findings of multiple nodules throughout the body, a diagnosis of IM with colorectal involvement was rendered. Cytogenetic studies performed on the subcutaneous nodule biopsy revealed a normal karyotype.

## Discussion

Up to 40% of the patients with IM have visceral lesions that are invariably present at birth [2]. The most common visceral sites are the lung, heart, gastrointestinal tract, pancreas, and rarely central nervous system [2,7,8]. Approximately 20 cases of gastrointestinal involvement have been reported in the literature. Lesions are usually found in the jejunum and ileum but have been described in the entire gastrointestinal tract from the esophagus to the rectum [5-7,9,10]. Most of these cases were diagnosed on the first day to 14 months of age and most cases had systemic diseases primarily involving the skin, musculoskeletal system and lung. Clinical manifestations of gastrointestinal involvement include diarrhea, poor feeding, weight loss, vomiting, hematochezia, decreased stool formation, and rarely bowel obstruction, perforation or intussusception [3-5,11]. The lesional nodules can be found on the serosal surface, in the subserosa, muscularis propria, submucosa, or mucosa [5-7,9,10].

There has been only one IM case that presented with intussusception reported in the literature [11]. The patient was a 5-week-old full-term boy who was noted to have well-demarcated, ~0.5 cm, and reddish marks all over his body. He presented with acute bile-stained vomiting accompanied by intermittent screaming, abdominal distension, tenderness in the left iliac fossa, and blood on rectal examination. Laparotomy revealed an ileocolic intussusception with a 1 cm polyp in the ileum. An anorectal polyp was subsequently found and excised. Histologic examination of these two polyps as well as a biopsy of the skin lesion showed spindle cell proliferation, morphologically consistent with IM.

Our case is unique in that colorectal IM caused rectal prolapse, which has not been documented in previously reported cases. Colo-colonic intussusception observed in our case is also a unique finding because the sigmoid colon is an unusual location for intussusception to occur and IM nodules apparently served as the lead. Another interesting observation in our case is that visceral IM was only detected in the colorectum but spared the small intestine and the most frequently involved visceral site, the lungs, at least by imaging studies. The etiology for this unusual IM case remains unclear but it is interesting to note that the other twin was completely devoid of any myofibroblastic lesions.

Because of its rarity, the histologic features of intestinal IM are poorly defined. An earlier case report of gastrointestinal multicentric IM described the histologic features as diffuse and nodular mucosal and submucosal fibrosis, small fascicles of plump fibroblast-like cells, decreased mucosal glands, and sparing of the muscularis mucosae [7]. The histologic findings in our case are similar to these descriptions except that the colonic crypts do not appear to be significantly decreased in number. Rather, the crypts are distorted and compressed, and the epithelial cells exhibit reactive and regenerative changes. In addition, surface erosions and mixed inflammatory cell infiltration are invariably present in every nodule in our case. Given these histologic findings, it is conceivable that the diagnosis of IM can be difficult to achieve if an individual mucosal nodule or polyp is biopsied, particularly in the lack of pertinent clinical history. Potential differential diagnoses may include inflammatory polyp, fibroblastic polyp, benign peripheral nerve sheath tumor, juvenile polyp, leiomyoma, gastrointestinal stromal tumor, inflammatory myofibroblastic tumor, inflammatory fibroid polyp, and ischemic enterocolitis. Some of these possibilities can be readily excluded based on the age of the patient, such as gastrointestinal stromal tumor, whereas others may require immunohistochemical studies. The importance of effective communication between pathologists and clinicians to find out extragastrointestinal manifestations cannot be overemphasized.

It has been shown that solitary and multicentric IM in the absence of visceral involvement bears an excellent prognosis. The lesions may either undergo spontaneous regression or be cured by simple local excision. The prognosis is poor in infants with visceral lesions [12]. This is particularly true of patients with lung involvement [8]. However, rare cases have shown spontaneous regression of the visceral lesions [1,13]. It is uncertain about the prognosis for patients with intestinal IM only in the absence of involvement of other visceral organs because of the rarity of the cases. Our patient has been doing well for 6 months postoperatively, but long-term follow-up to watch for the development of additional complications of visceral lesions is warranted.

## Consent

Written informed consent was obtained from the patient' parents for publication of this case report and accompanying images. A copy of the written consent is available for review by the Editor-in-Chief of this journal.

## Competing interests

The authors declare that they have no competing interests.

## Authors' contributions

DD performed the histologic examination of the surgical specimens, did literature search and wrote the manuscript. PKF collected clinical data and critically read the manuscript. HLW performed the histologic examination of the surgical specimens, did literature search, and edited the manuscript. All authors have read and approved the final manuscript.
